# Dendritic cell-based vaccine prolongs survival and time to next therapy independently of the vaccine cell number

**DOI:** 10.1186/s13062-022-00318-w

**Published:** 2022-02-23

**Authors:** Simon Hawlina, Helena H. Chowdhury, Tomaž Smrkolj, Robert Zorec

**Affiliations:** 1 Clinical Department of Urology, University Medical Centre Ljubljana, 1000 Ljubljana, Slovenia; 2 Department of Surgery, Faculty of Medicine, University of Ljubljana, 1000 Ljubljana, Slovenia; 3 Laboratory of Cell Engineering, Celica Biomedical, 1000 Ljubljana, Slovenia; 4 Laboratory of Neuroendocrinology – Molecular Cell Physiology, Institute of Pathophysiology, Faculty of Medicine, University of Ljubljana, Zaloska 4, 1000 Ljubljana, Slovenia

**Keywords:** Dendritic-tumor hybridoma vaccine, Autologous cell therapy, Immunotherapy, Castration-resistant prostate cancer

## Abstract

In 2009, new EU legislation regulating advanced therapy medicinal products (ATMPs), consisting of gene therapy, tissue engineering and cell-based medicines, was introduced. Although less than 20 ATMPs were authorized since that time, the awarding of the Nobel Prize for Physiology or Medicine in 2018 revived interest in developing new cancer immunotherapies involving significant manipulation of the patient's own immune cells, including lymphocytes and dendritic cells. The lymphocytes are mainly thought to directly affect tumour cells, dendritic cells are involved in indirect mechanisms by antigen presentation to other leukocytes orchestrating the immune response. It is the latter cells that are the focus of this brief review. Based on the recent results of our study treating patients with castration-resistant prostate cancer (CRPC) with an immunohybridoma cell construct (termed aHyC), produced by electrofusion of autologous tumour and dendritic cells, we compare their effectiveness with a matched documented control group of patients. The results revealed that cancer-specific survival and the time to next in-line therapy (TTNT) were both significantly prolonged versus controls. When patients were observed for longer periods since the time of diagnosis of CRPC, 20% of patients had not yet progressed to the next in-line therapy even though the time under observation was ~ 80 months. Interestingly, analysis of survival of patients revealed that the effectiveness of treatment was independent of the number of cells in the vaccine used for treatment. It is concluded that autologous dendritic cell-based immunotherapy is a new possibility to treat not only CRPC but also other solid tumours.

## Background

When the EU regulation for advanced therapy medicinal products (ATMPs), consisting of gene therapy medicinal products, tissue-engineered products and cell therapy medicinal products (CTMPs), was introduced in 2009, this was considered to facilitate the innovation of better medicines, offering potential treatment opportunities for diseases that currently have limited or no effective therapeutic options. Although ATMPs have gained considerable interest in the last decade or so, this has been associated with new challenges, mainly how to consider regulatory approaches that have been adopted in the past through the development of small-molecule-based medicines [1]. This is particularly relevant to using somatic cells, exposing them to substantial manipulation ex vivo and then returning them into the patient for treatment. Discussions pertinent to the use of CTMPs have frequently revisited the question whether the effectiveness of these therapies depends on the quantity of cells (cell dose); for example, whether a single dose of cells or repetitive administration of cells is needed, highlighting the fact that the mechanisms of action of these cells are mostly unknown, at least in the case of treating heart failure with stem cells [2]. Moreover, this question is also relevant for understanding of how cell-based medicines act in cancer cell-based immunotherapy, which is based on the power and specificity of the immune cells for the treatment of malignancy [3].

The immune system has a potential capability to recognize and attack cancer cells through the process of “immuno-editing”, including elimination, equilibrium, and escape phases [4]. Tumour cells may escape immune recognition using several mechanisms that are usually associated with a protective function of healthy tissues from autoimmune interactions. These consist of inefficient processing and presentation of tumour antigens, upregulation of negative costimulatory ligands that mediate T cell anergy [5], expansion of regulatory cells, and production of “immunosuppressive molecules”, including Fas ligand [6], transforming growth factor β [7] and the potentially reversible immunosuppressive enzyme indolamine-2,3-dioxygenase [8, 9]. In addition, tumour cells can directly escape T cell recognition through downregulating major histocompatibility complex (MHC) class I or disabling other components of antigen presentation [10]. To combat cancer by cell-based-therapies, the intrinsic capacity of dendritic cells (DCs) to augment antitumor immune effector cells, such as tumour antigen-specific cytotoxic T lymphocytes and natural killer (NK) cells, can be harnessed [11].

In this article, we first review how DCs have been used to treat prostate cancer. Then, we focus on the recently conducted clinical trial involving cell-based immunotherapy of castration-resistant prostate cancer (CRPC) [12], in which autologous immunohybridomas (aHyC), produced by electrofusion of the patient's own tumour and DCs, were used [13, 14]. Previously, these immunohybridomas were shown to augment the cytotoxic immune cell capacity in vitro [15], a mechanism playing a role in the cell-based therapy of cancer. We then compare cancer-specific survival (CSS) and time to next in-line therapy (TTNT) of patients treated with the DC-based vaccine (aHyC) in a recent clinical trial [12] with patients in a documented control group. Finally, we consider the question of the cell dose by comparing the CSS and TTNT of patients with CRPC with the number of cells used in the vaccine.

### Prostate cancer, immunotherapy and dendritic cells

**Fig. 1:**
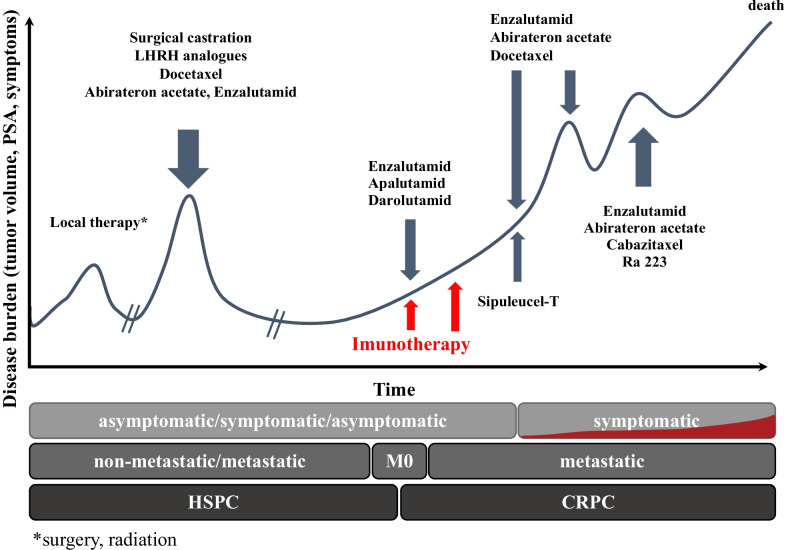
Stages of prostate cancer leading to castration-resistant prostate cancer (CRPC). Time course of the development of CRPC depicted as arbitrary tumour volume (ordinate) as a function of time (arbitrary units). About 28% of patients with prostate cancer develop CRPC. Arrows indicate time points of standard treatments (Table 1). The red arrows indicate a suitable time points for cell-based immunotherapy to be as early as possible. The asterisk denotes local therapy radiotherapy/surgery. HSPC, hormone-sensitive prostate cancer [55]

Prostate cancer (PCa), a common malignancy in men [16], can advance to the incurable CRPC. Not long ago, docetaxel chemotherapy was the only effective treatment for CRPC [17, 18]. After the approval of several new treatments, including the alpha emitter radium-223 [19], second-line taxane cabazitaxel [20, 21], and the cell-based vaccine sipuleucel-T [22], patient survival was shown to be improved by up to 7 months [23–26]. On the basis of five phase 3 clinical trials (COU-AA-302, PREVAIL, PROSPER, SPARTAN and ARAMIS), in which improvements in metastasis-free survival and patient survival were shown, new androgen inhibitors, abiraterone acetate, enzalutamide, apalutamid, and darolutamid [27–31] were used. Figure 1 depicts the time course of CRPC and the time points of various therapies, standard and recently introduced. Table 1 presents data on the known mechanisms, efficacy, and toxicity of individual therapies, both currently approved and new immunotherapy for the treatment of CRPC.

**Table 1:** Current standard (approved) therapies and tested new immunotherapies for castration-resistant prostate cancer

Name of therapy	Standard, approved (FDA or EMA), yes/no	Mechanism of action	Efficacy (patient survival, other)	Toxicity/side effects	References
Abiraterone acetate	Yes	Androgen synthetic inhibitor	mOS abiraterone acetate + prednisone versus placebo + prednisone group: 34.7 months (95% CI, 32.7–36.8) versus 30.3 months (28.7–33.3); HR, 0.81 (95% CI, 0.70–0.93; *P* < 0.0033)	Yes. Abiraterone acetate + prednisone versus placebo: grade 3–4: hypertension 5% versus 3%; hypokalaemia 2% versus 2%; serious AEs of any grade: 38% versus 27%; serious AE hypokalaemia: <1% versus 0; treatment-related deaths: 0% versus 0%	[56]
Enzalutamide	Yes	Androgen receptor inhibitor	mOS enzalutamide versus placebo group: 67.0 months (95% CI, 64.0–NR) versus 56.3 months (95% CI, 54.4–63.0); HR, 0.73 (95% CI, 0.61–0.89; *P* = 0.001)	Yes. Grade ≥ 3 the exposure-adjusted rate of AEs in the enzalutamide versus placebo group: 17/100 patient-years versus 20/100 patient-years. Most frequently reported AE in the enzalutamide group: fatigue and musculoskeletal events	[29, 57]
Apalutamid	Yes	Androgen receptor inhibitor	mMFS apalutamide versus placebo group: 40.5 months versus 16.2 months; HR for metastasis or death, 0.28 (95% CI, 0.23–0.35; *P* < 0.001). Time to symptomatic progression was significantly longer with apalutamide than with placebo; HR, 0.45 (95% CI, 0.32–0.63; *P* < 0.001)	Yes. The rate of AE leading to discontinuation of the trial regimen: apalutamide versus placebo group: 10.6% versus 7.0%. AEs occurred at a higher rate with apalutamide than with placebo: rash (23.8% versus 5.5%), hypothyroidism (8.1% versus 2.0%), fracture (11.7% versus 6.5%)	[31]
Darolutamid	Yes (FDA)	Androgen receptor inhibitor	3-year OS darolutamide versus placebo: 83% (95% CI, 80–86) versus 77% (95% CI, 72 to 81). The risk of death was significantly lower, by 31%, in the darolutamide group than in the placebo group; HR for death, 0.69 (95% CI, 0.53–0.88; *P* = 0.003). Darolutamide was associated with a significant benefit with respect to the time to first symptomatic skeletal event and the time to first use of cytotoxic chemotherapy	No. The incidence of AEs after the start of treatment was similar in the two groups; no new safety signals were observed	[30, 58]
Docetaxel	Yes	Chemotherapeutic drug	Docetaxel + estramustine versus mitoxantrone and prednisone: mOS: 17.5 months versus 15.6 months, *P* = 0.02; HR, 0.80 (95% CI, 0.67–0.97). mPFS: 6.3 months versus 3.2 months (*P* < 0.001). ≥ PSA decline: 50% versus 27% (*P* < 0.001). Objective tumour responses: 17% versus 11% (*P* = 0.30)	Yes. Grade 3 or 4 more common in docetaxel + estramustine versus mitoxantrone + prednisone group: neutropenic fevers, (*P* = 0.01), nausea and vomiting (*P* < 0.001), cardiovascular events (*P* = 0.001)	[59]
Cabazitaxel	Yes	Chemotherapeutic drug	Cabazitaxel versus androgen-signalling-targeted inhibitor: imaging-based progression or death: 73.6% versus 80.2%; HR, 0.54 (95% CI, 0.40–0.73; *P* < 0.001). mPFS (imaging-based): 8.0 versus 3.7 months. mOS: 13.6 versus 11.0 months; HR, 0.64 (95% CI, 0.46–0.89; P = 0.008). mPFS 4.4 versus 2.7 months; HR, 0.52 (95% CI, 0.40–0.68; *P* < 0.001). PSA response: 35.7% versus 13.5% (*P* < 0.001). Tumour response: 36.5% versus 11.5% (*P* = 0.004)	Yes/no. Cabazitaxel versus androgen-signalling-targeted inhibitor: grade ≥ 3 AE: 56.3% versus 52.4%; no new safety signals were observed	[60]
Olaparib	Yes (FDA)	PARP inhibitor	Olaparib versus enzalutamide or abiraterone: rPFS: 7.4 months versus 3.6 months (HR, 0.34; 95% CI, 0.25–0.47; *P* < 0.001), confirmed ORR 33% versus 2% (OR 20.86, 95% CI, 4.18–379.18: *P* < 0.001). mOS: 18.5 months versus 15.1 months (in pts with BRCA1/2, ATM alterations), (HR, 0.64, 95% CI, 0.43–0.97; *P* = 0.02)	Yes. The incidence of AEs of grade 3 or higher was higher with olaparib than with the control treatment. The most common AEs: anemia, nausea, and fatigue or asthenia with olaparib and fatigue or asthenia with the control treatment. A total of 11 cases of pulmonary embolism (4% of patients) were reported in the olaparib group, as compared with 1 (1%) in the control group; none were fatal	[61]
Sipuleucel-T	Yes (FDA)	Cell-based immunotherapy	Sipuleucel-T versus placebo group: relative risk reduction of death, 22%; HR, 0.78 (95% CI, 0.61–0.98; *P* = 0.03). mOS: 25.8 versus 21.7 months. 3-year survival probability: 31.7% versus 23.0%; HR, 0.77 (95% CI, 0.61–0.97; *P* = 0.02). Immune responses to the immunizing antigen were observed in patients who received sipuleucel-T	Yes. AEs more frequently reported in the sipuleucel-T group: chills, fever, headache	[22, 62]
Ipilimumab	No	Immune check-point inhibitor	No. Ipilimumab versus placebo: mOS: 28.7 months (95% CI, 24.5–32.5) versus 29.7 months (95% CI, 26.1–34.2); HR, 1.11 (95.87% CI, 0.88–1.39; *P* = 0.367). mPFS: 5.6 versus 3.8 months; HR, 0.67 (95.87% CI, 0.55–0.81). PSA response rate: 23% versus 8%	Yes. Grade 3–4 treatment-related AEs reported in ≥ 10% of ipilimumab-treated patients: diarrhoea (15%). Ipilimumab versus placebo: deaths, 2% versus 0; immune-related grade 3–4 AEs: 31% versus 2%	[32]
aHyC	Non-routine, hospital exemption	DC-tumour immunohybridoma	mOS: 58.5 months (95% CI, 38.8–78.2). mCSS: 75.7 months (95% CI, 41.1–110.4)	No. Only grade 1 treatment-related AEs (e.g., asthenia, pelvic pain, rush)	[12]
Pembrolizumab	Yes (FDA)	Immune check-point inhibitor	mrPFS 2.1 months (95% CI, 2.1–2.2). mOS 9.6 months (95% CI, 7.9–12.2)	Yes. Grade 3–5 treatment-related AEs: 15% patients. Discontinuation of pembrolizumab because of a treatment-related AE: 5% patients. Deaths due to treatment-related AEs: 0.8% (*n* = 2, pneumonitis and sepsis). The most common treatment-related AEs: fatigue, diarrhoea, decreased appetite. Immune-mediated AEs and infusion reactions: 16% patients (grade 3–5 in 6% patients; led to discontinuation in 2% patients and death in < 1%). The most common immune-mediated AEs were colitis, hyperthyroidism, hypothyroidism, pneumonitis, and severe skin reactions	[33, 63–65]

*AE* adverse event, *CI* confidence interval, *FDA* US Food and Drug Administration, *EMA* European Medicines Agency, *HR* hazard ratio, *m* median, *MFS* metastases-free survival, *NR* not reached, *ORR* objective response rate, *OR* odds ratio, *OS* overall survival, *PFS* progression-free survival, *rPFS* radiographic progression-free survival, *PSA* prostate-specific antigen

Advances in cancer immunotherapy and especially the awarding of the Nobel Prize for Physiology or Medicine in 2018 to J.P. Allison and T. Honjo "for their discovery of cancer therapy by inhibition of negative immune regulation", have facilitated interest in PCa immunotherapy. However, as seen in Table 1, ipilimumab, an immune-checkpoint blocker failed to demonstrate survival benefit in patients with CRPC [32] and pembrolizumab was approved by the US Food and Drug Administration for all mismatch repair-deficient cancers or those with unstable microsatellite status, which is a rare finding in PCa [33]; hence, sipuleucel-T appears to be the only approved immunotherapy for CRPC [22]. The proposed mechanism of action of sipuleucel-T, a cell-based vaccine, is induction of antigen-specific immune responses against prostatic acid phosphatase on PCa cells [34].

In previous immunotherapy clinical trials of prostate cancer treatment, several arrangements were used to address antigen presentation, ranging from a completely general strategy (most feasible in terms of vaccine production), where one antigen was used to modulate the immune system in all patients, to others with more individualized approaches (more complex and less feasible in terms of production). The general, one-for-all approach was used in the PROSTVAC trial, a viral vector-based immunotherapy consisting of recombinant poxviruses expressing prostate-specific antigen (PSA), together with three immune-enhancing costimulatory molecules, aiming to induce PSA-specific T cell responses, eventually killing PSA-expressing tumour cells [35]. The GVAX-PCa trial used a vaccine consisting of a mixture of two irradiated prostate cancer cell lines, LNCaP and PC-3, with a constitutively expressed granulocyte–macrophage colony-stimulating factor (GM-CSF), an immune cell activator [36]. In the PROSTVAC and GVAX trials, in which the vaccine was administered subcutaneously/intradermally, these treatments tended to improve median overall survival [24]. As most prostate cancer cells express prostatic acidic phosphatase (PAP) [37], this has been used to incubate the patient’s isolated cells on an autologous basis to produce sipuleucel-T [22], which is more production intensive than the approaches mentioned earlier. Briefly, antigen-presenting cells extracted from autologous peripheral blood mononuclear cells were activated ex vivo with a recombinant fusion protein (PA2024) consisting of PAP fused to GM-CSF. The processed cells were then infused into the patient, resulting in a 4.1-month improvement in median survival, however with more side effects than in patients receiving placebo [22]. However, it is unclear whether sipuleucel-T acts via priming of naive T cells through antigen-presenting DCs, because it consists of less than 20% of DC markers [38].

DCs are able to activate both naive and memory T cells and appear an ideal target for augmenting antitumor immune responses [39]. Thus, vaccination with enriched, activated DCs may be a more potent immunotherapy strategy than the afore-mentioned approaches. Consistent with this, vaccination by blood-derived DCs, stimulated with protamine/mRNA and loaded with three tumour-associated antigens (NY-ESO-1, MAGE-C2 and MUC) resulted in more frequent detection of tetramer/dextramer-positive (dm^+^) and interferon-gamma (IFN-γ)-producing antigen-specific T cells in skin biopsy samples of patients with radiologic non-progressive disease versus patients with CRPC with progressive disease; the overall median radiologic progression-free survival was 9.5 months [40].

Instead of selecting one [22] or a few [40] effective tumour antigens that may elicit sufficient immunologic response in DC-based vaccines to treat patients with CPRC, whole tumour cells were used as the source of tumour antigens. This was achieved by electrofusing tumour and DCs to produce hybridomas, an approach developed previously and evaluated by confocal microscopy and flow cytometry [13]. Antigen presentation involves late endocytic compartments, containing MHC class II molecules, therefore heterotypic vesicle fusion is needed to deliver antigens to MHC class II molecules in hybridomas. It was shown that fusion of late endocytic compartments also takes place in hybridomas and that the efficiency of this approach, measured as an enhanced in vitro cytotoxic T cell response, is stronger if a higher percentage of fused late endocytic compartments is present in the cell population of electrofused hybridoma cells [14, 15]. The advantage of such hybridomas over other forms of DC vaccines [41] is their presentation capacity of both known and yet unknown tumour-associated antigens to T lymphocytes and other immune cells.

**Fig. 2:**
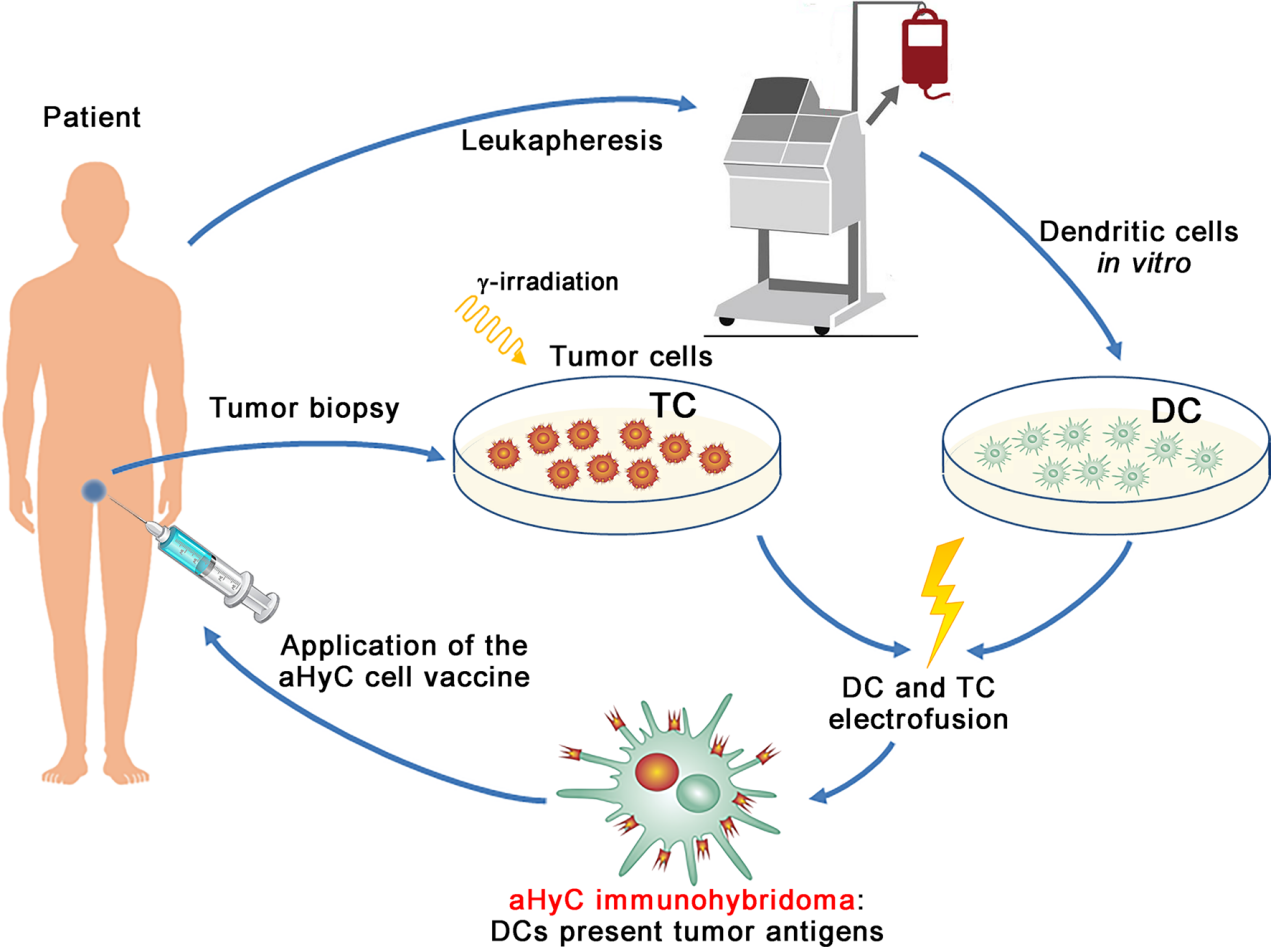
The procedures used in the clinical trial treating castration-resistant prostate cancer (CRPC) [12]. Biopsy samples of the prostate were taken and a suspension of tumour cells (TC) was produced in the GMP facility. Monocytes were harvested from the same patient by leukapheresis to produce dendritic cells (DC) in the lab. These were then electrofused with TCs to obtain immunohybridomas, aHyC, which were applied subcutaneously into the patient four times

Recently, these completely autologous dendritic-tumour immunohybridoma cells, termed aHyC, produced as described [13], were used to treat chemotherapy-naive patients with CRPC in a phase 1/2, double-blind, cross-over clinical trial [12]. The procedure for treating patients with CPRC with aHyC, from performing the biopsy, harvesting monocytes by leukapheresis, then using electrofusion to generate immunohybridomas and using subcutaneous injection of the cell suspension, is shown in Fig. 2. This trial tested the feasibility, assessed the safety and quality of life and evaluated clinical and immunological outcomes and overall survival (OS), with a median OS of 58.8 months [12]. Monitoring several leukocyte populations before and after vaccination, the results revealed that survival of patients with CRPC was inversely correlated with changes in peripheral blood CD56^bright^ CD16^−^ natural killer (NK) cells [12]. These cells are considered immunoregulatory cytokine-producing cells, which on appropriate activation (proinflammatory cytokines IL-2 and IL-15), can become cytotoxic [42]. In peripheral blood, CD56^bright^CD16^−^ NK cells represent a small fraction (typically around 10%) of all NK cells [43]. An increase in the fraction of CD56^bright^CD16^−^ NK cells was observed in the placebo and aHyC arms. However, in the aHyC-first application group, this increase was significantly reduced, indicating that this may contribute to the beneficial clinical outcome recorded [12], because these regulatory NK cells may contribute to a mechanism by which tumours can evade the host immune response [44, 45]. Consistent with this in advanced malignancies, such as melanoma and breast cancer, an increase in the fraction of these cells was found to be associated with a prometastatic function of peripheral blood CD56^bright^CD16^−^ NK cells [46, 47]. However, because only a few studies addressed changes in a circulating CD56^bright^CD16^−^ NK cell population in cancerous conditions previously [46–52], this needs to be readdressed in the future. The IMPACT trial (sipuleucel-T) has been subject to criticisms [53], including that there was no change in progression-free survival, no significant impacts on PSA, tumour burden, symptoms, or pain. Without a meaningful impact on surrogate endpoints, it is hard to understand and explain the observed improvement in OS. In addition, it would be beneficial to identify a marker in peripheral blood that can predictably inform clinicians and patients about the efficacy of the vaccine after treatment. Interestingly, changes in the percentage of peripheral blood CD56^bright^CD16^−^ NK cell population could be a biomarker for monitoring the effectiveness of the treatment, predicting the prognosis and adjusting the therapy as soon as possible if necessary.

To further verify the effectiveness of aHyC therapy in the treatment of patients with CRPC [12], we asked whether the TTNT is altered and whether the efficacy of aHyC treatment depends on the dose of cells used in the vaccine.

### Prolongation of survival and TTNT in patients with CRPC treated with aHyC

To learn whether the application of aHyC affected the clinical outcome of patients, we determined the CSS and TTNT (Fig. 1), both measures of effectiveness of aHyC treatment. We compared these parameters in relation to a matched control group of patients who did not receive aHyC (Table 2). To compare these parameters in both groups, both CSS and TTNT were determined in months from the time of diagnosis of CRPC to the cut-off date (30 September 2021) or the patient’s death. We included all patients who received aHyC vaccine (aHyC group, *n* = 19) and a control group (*n* = 21). At the cut-off date, the incidence of any cause of death was 58% (11 patients) within the aHyC group and 90% (19 patients) in the documented control group. Both groups were comparable for age at CRPC diagnosis (74 and 72 years) and other characteristics (Table 2).

**Table 2:** Patient characteristics in the aHyC group (all patients who received aHyC) and in the documented control group

	aHyC treatment group	Documented control group
Number of patients	19	21
Age (years) at CRPC diagnosis, median (IQR)	74 (69–81)	72 (69–75)
Follow-up (months), median (IQR)	65 (35–81)	50 (41–59)
Deaths, *n* (%)	11 (58)	19 (90)
PSA at CRPC diagnosis, ng/mL		
Median (IQR)	7 (4–14)	11 (6–14)
Mean ± SEM	14 ± 6	13 ± 3
Gleason score, *n* (%)		
8–10	16 (84)	12 (57)
6–7	3 (16)	9 (43)
Median (IQR)	9 (9–9)	8 (7–9)
Metastases at CRPC*, *n* (%)		
No metastases	12 (63)	15 (71)
Oligometastases (≤ 3)	3 (21)	6 (29)
Polymetastases (≥ 4)	4 (16)	0
Site of metastases*, n (%)		
Bone	4 (21)	4 (19)
Lymph node	0	2 (10)
Bone + lymph node	3 (16)	0
Visceral	0	0
Next-in-line treatment (docetaxel, enzalutamide, abiraterone acetate) up to 30 September 2021		
Yes, n (%)	13 (68)	21 (100)
TTNT (months), median (HR; 95% CI)	28 (0.31; 0.15–0.63)	16 (3.25; 1.59–6.64)

*aHyC* autologous hybridoma cell, *CI* confidence interval, *CRPC* castration-resistant prostate cancer, *HR* hazard ratio, *IQR* interquartile range, *PSA* prostate-specific antigen, *TTNT* time to next therapy

*Metastases were determined with routinely performed nuclear medicine bone scan and computed tomography of thorax and abdomen or ^18^F choline positron emission tomography-computed tomography

**Fig. 3:**
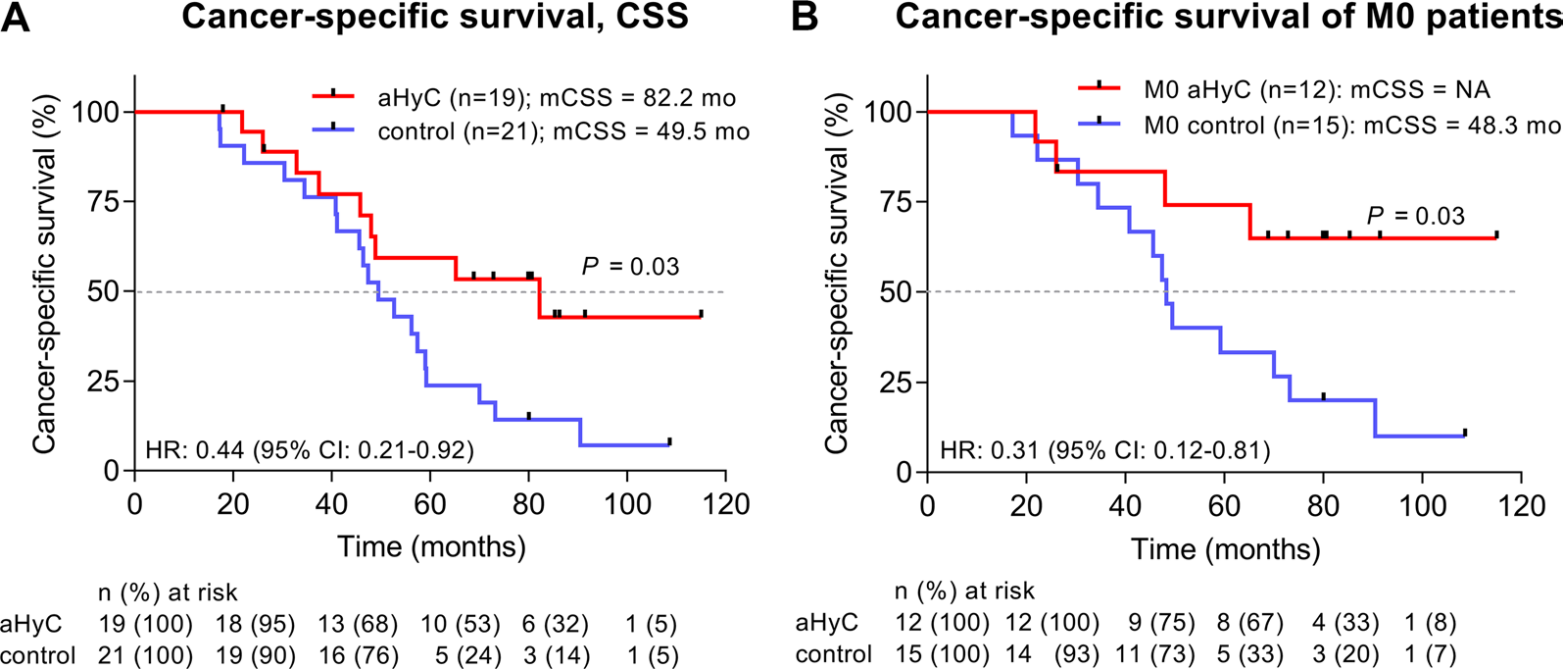
Cancer-specific survival (CSS). **A** CSS after diagnosis of castration-resistant prostate cancer (CRPC) was significantly prolonged (*P* = 0.03) by 32.7 months in patients who received aHyC (red, 82.2 months) compared with patients in the documented control group who did not receive aHyC (blue, 49.5 months). **B** CSS was further compared only in patients with non-metastatic (M0) CRPC. Median CSS survival was not reached in patients who received aHyC (red line), and it was significantly shorter (*P* = 0.03) in patients in the documented control group who did not receive aHyC (blue line, 48.3 months). Black dots on the lines represent censored events of specific survival. The common starting point for both groups of patients was the diagnosis of CRPC; cut-off date was 30 September 2021. The tables below the graphs show the number and proportion of patients in both groups who are still at risk at individual time points on the graphs. NA, median value not yet reached; M0, non-metastatic CRPC

From the time of diagnosis of CRPC, CSS was prolonged by 32.7 months (*P* < 0.05) in patients who received aHyC (82.2 months) compared with patients in the documented control group who did not receive aHyC (49.5 months; Fig. 3A). In patients with non-metastatic CRPC (M0; Fig. 3B) who received aHyC (note that the median value is not reached), CSS was much longer (*P* = 0.03) than in patients in the documented control group who did not receive aHyC (48.3 months). The considerably prolonged CSS in M0 aHyC-treated patients compared with CSS recorded in all, metastatic (M1) and M0 patients treated with aHyC (Fig. 3A) clearly suggests that prostate cancer vaccines may be more beneficial when given early, at the stage of M0, because the immune system has time to mount a response, the disease burden is low and before immune system evasion by the tumour [54]. Moreover, it is likely that sourcing tumour antigens by biopsy from the prostate is relatively complete in non-metastatic disease, whereas in metastatic patients, tumour antigens in the metastases differ significantly from tumour antigens in the primary tumour in the prostate, and thus in the vaccine. These results indicate that patients with non-metastatic CRPC will benefit best from treating with immunotherapy aHyC.

**Fig. 4:**
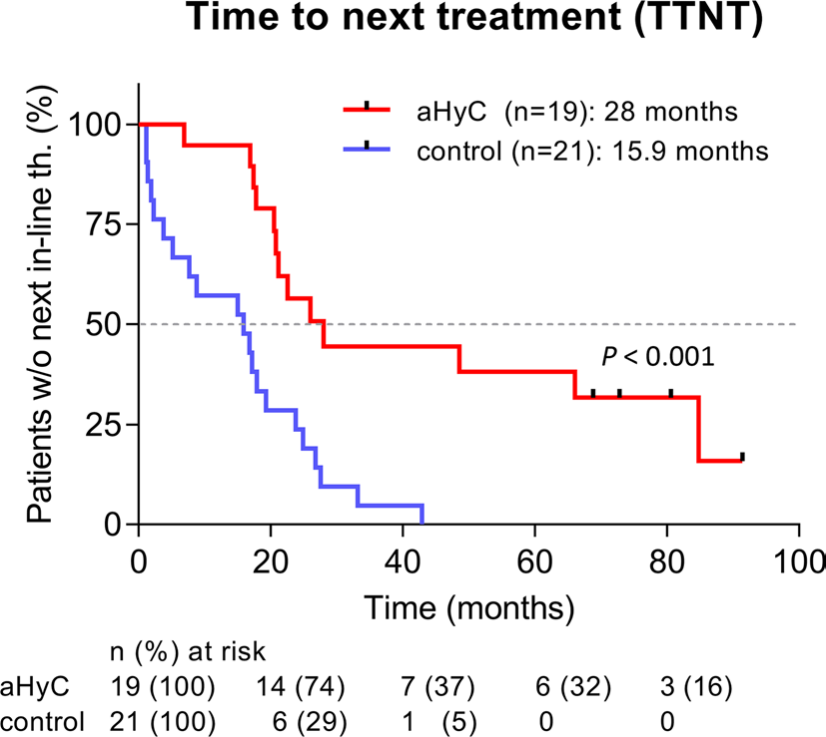
Time to next therapy (TTNT) from the time of diagnosis of castration-resistant prostate cancer (CRPC). The ordinate denotes the percentage of patients without next in-line therapy. The red curve represents patients who received aHyC therapy; the median TTNT (mTTNT) was 28 months, significantly longer (*P* < 0.001) than the time recorded in control patients (blue curve; 15.9 months), yielding a prolongation of 12.1 months without the need for the next in-line therapy by the aHyC application. In both groups, TTNT was determined by taking into account the time of CRPC diagnosis; data were analysed considering the cut-off date 30 September 2021. Black marks on the red curve indicate censored events (patients who did not receive the next in-line therapy). The table below the plot indicates the number and percentage of patients in both groups who have not yet received any next in-line standard therapy at time points in months on the plot

Interestingly, TTNT was determined as the time from CRPC diagnosis to the beginning of the next in-line standard treatment (i.e., docetaxel, abiraterone acetate, enzalutamide; Fig. 1) or to the cut-off date in both groups. The same criteria were used for the introduction of the next in-line treatment in patients in the aHyC group and in the control group (Table 2). The median TTNT in the aHyC group was 12.1 months longer (28.0 months, hazard ratio, 0.31 (95% confidence interval, 0.15–0.63); *P* < 0.001) than that in the control group (15.9 months; Fig. 4). Overall, these results indicate that aHyC treatment is beneficial for patients by prolonging CSS and TTNT. To better understand the mechanism of this process, we looked next at the cell number in the vaccine used to treat patients.

### Independent relationship between survival or TTNT and the cell number in the vaccine

**Fig. 5:**
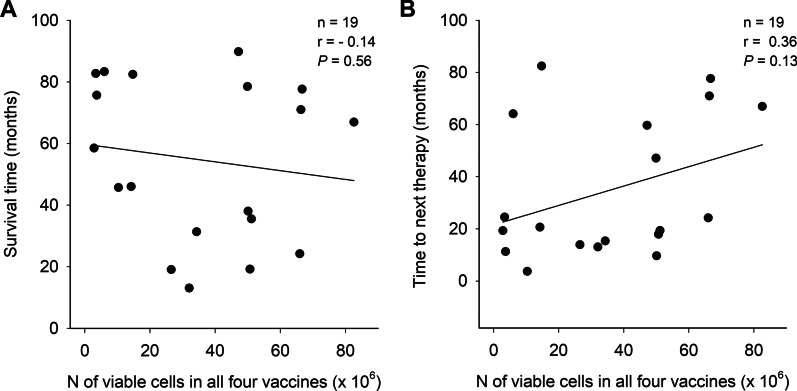
Independence of survival and time to next therapy (TTNT) from the number of cells in vaccines. **A** The ordinate denotes the survival of patients with castration-resistant prostate cancer (CRPC) treated with aHyC and the abscissa shows the number of cells in the vaccination procedure in each of the patients. Survival was determined from the time of first application (aHyC or placebo) until death or the cut-off date (30 September 2021). **B** The ordinate denotes the time (in months) to TTNT from the start of the clinical trial and the abscissa shows the number of cells in the vaccination procedure for each of the 19 patients. TTNT was determined from the time of first application (aHyC or placebo) until the next in-line therapy or the cut-off date (30 September 2021). Pearson correlation coefficients (*r*) show that there is no significant relationship between the survival of patients with CRPC or TTNT and the number of cells in the vaccines

In a recent clinical study, it was revealed that the survival of patients with CRPC was negatively correlated with the change in the percentage of the peripheral blood CD56^bright^CD16^−^ NK cell population [12]. This indicates that subcutaneous injection of the aHyC cell vaccine induced an immune response, measured as a reduction in the subpopulation of NK cells. This raises the question of how this measured change was linked to the DC-based vaccination by aHyC? One way to address this issue is to determine whether the immune response and the survival or TTNT depended on the number of cells in the vaccine. Given that the amount of cells harvested in the prostate biopsies varied between patients [12], we asked whether the number of cells in the aHyC vaccine was related to the survival of patients. Figure 5A shows the relationship between the survival of patients as a function of the number of cells in the vaccination procedure. The Pearson correlation coefficient *r* is − 0.14. A similar non-significant *r* of 0.36 was found in the relationship between the TTNT and the number of cells in the vaccination treatment (Fig. 5B). One would expect that better survival or longer TTNT would be related to the cell dose, because this is expected in dose-dependent studies using small-molecule medicines. However, in cell-based therapy, especially when DCs are used to generate antigen presentation, perhaps a low number of cells or even just one is needed to generate a threshold-dependent effect in antigen presentation and indirectly affect tumour cells. This will have to be investigated further in the future.

## Conclusions

DCs have been used in clinical trials as a form of therapeutic vaccination of patients with cancer for over three decades, demonstrating that this approach is safe and can induce antitumor immunity. However, the clinical responses have been disappointing, because not all studies were designed primarily to measure survival [11]. As presented in this brief review, this has improved.

A recent study treating patients with CRPC revealed that survival of patients was negatively correlated to a subpopulation of NK cells [12], indicating that DC-based vaccines engage NK cells in the immune response, as considered previously [11]. Moreover, we show the relationship between survival and the dose of cells in the vaccine (Fig. 5). These data are important not only for understanding the mechanism of action of DC cell-based immunotherapy but also in view of the legislation and rules dealing with ATMPs, a key challenge in implementing regulatory approaches that have been adopted in the past through the development of small-molecule-based medicines [1]. For example, to validate the safety of new small-molecule-based medicines, it is imperative to carry out dose-escalation studies, but in cell-based advanced immunotherapy products, the mechanism of action may not be as simple as the interaction between a small molecule and its receptor. Here, we show that survival or TTNT is independent of the number of cells in the vaccine, pointing to the possibility that, in the case of DC-based vaccines, a minimal threshold number of cells is needed to elicit a treatment-related immune response. Therefore, this needs to be taken into account in preparing DC-tumour immunohybridoma vaccines in future clinical trial designs.

## Data Availability

Data generated and analyzed during the current study are available from the corresponding author on reasonable request. Clinical trial protocol is available at link: http://www.lnmcp.mf.uni-lj.si/Protocol.pdf.

## Abbreviations

AE: Adverse event
aHyC: Autologous hybridoma cell
ATMP: Advanced therapy medicinal products
CI: Confidence interval
CRPC: Castration-resistant prostate cancer
CSS: Cancer-specific survival
CTMP: Cell therapy medicinal products
DC: Dendritic cell
EU: European Union
FDA: US Food and Drug Administration
EMA: European Medicines Agency
GM-CSF: Granulocyte–macrophage colony-stimulating factor
GMP: Good manufacturing practice
HR: Hazard ratio
HSPC: Hormone-sensitive prostate cancer
IFN-γ: Interferon-gamma
IL: Interleukin
IQR: Interquartile range
M0: Non-metastatic
M1: Metastatic
m: Median
MFS: Metastases-free survival
MHC: Major histocompatibility complex
NA: Not applicable
NK: Natural killer
NR: Not reached
ORR: Objective response rate
OR: Odds ratio
OS: Overall survival
PAP: Prostatic acidic phosphatase
PCa: Prostate cancer
PFS: Progression-free survival
rPFS: Radiographic progression-free survival
PSA: Prostate-specific antigen
TC: Tumour cells
TTNT: Time to next in-line therapy
